# The value of eZIS analysis of Tc-99m ECD SPECT on identifying cerebellar hypoperfusion in a patient with superficial siderosis: A case report

**DOI:** 10.1097/MD.0000000000005416

**Published:** 2016-11-28

**Authors:** Cheng-Yu Wei, Tai-Yi Chen, Ian Shih, Pai-Yi Chiu, Guang-Uei Hung, Hiroshi Matsuda

**Affiliations:** a Department of Neurology, Chang Bing Show Chwan Memorial Hospital, Changhua; b Department of Exercise and Health Promotion, College of Education, Chinese Culture University, Taipei; c Department of Radiology, Chang Bing Show Chwan Memorial Hospital, Changhua; d Department of Neurology, Cheng Ching Hospital Chung Kang Branch, Taichung; e Department of Nuclear Medicine, Chang Bing Show Chwan Memorial Hospital, Changhua; f Department of Biomedical Imaging and Radiological Science, China Medical University, Taichung, Taiwan; g Integrative Brain Imaging Center, National Center of Neurology and Psychiatry, Tokyo, Japan.

**Keywords:** cerebral perfusion, ECD, eZIS, SPECT, superficial siderosis

## Abstract

**Introduction:**

Brain perfusion single photon computed tomography (SPECT) is a functional imaging modality and has been widely utilized in evaluation of various kinds of neurological disorders. Easy z-score imaging system (eZIS) is a computer-assisted statistical analysis, based on the comparison with age-classified ethyl cysteinate dimer (ECD) normal database, which provides objectively interpretation of Tc-99m ECD brain perfusion SPECT.

Here we presented a 64-year-old male with dizziness, spin sensation, nausea, and vomiting in the emergency room, and brain computed tomography scan showed only small hypodensity lesion in cerebellum. Tc-99m ECD SPECT was performed for evaluating occult cerebral ischemia, infarction, and/or degeneration, but no remarkable abnormality could be identified by experienced readers on conventional display. The result of eZIS showed remarkable hypoperfusion in cerebellum and mild hypoperfusion in bilateral frontal and parietal lobes. Magnetic resonance imaging (MRI) confirmed severe atrophy of anterior cerebellar lobe. In addition, MRI showed diffuse hypointensity signals along with cerebrospinal fluid spaces, especially those areas with hypoperfusion on SPECT, compatible with typical appearances of superficial siderosis.

**Conclusion:**

This presented case demonstrates the value of software analysis with eZIS on enhancing the diagnostic value of brain perfusion SPECT for detecting brain lesions at an uncommon location due to a rare disease.

## 1 Introduction

As a functional neurological imaging modality, brain perfusion single photon computed tomography (SPECT) is useful for evaluations of cerebrovascular disease, epileptic disorder, and neurodegenerative disease.^[1–3]^ However, the visual interpretations of brain SPECT were less intuitive in comparison with anatomical images (such as computed tomography [CT] or magnetic resonance imaging [MRI]), more subjective and less reproducible.^[4]^ In order to overcome these drawbacks, computer-assisted stereotactic approaches, such as 3-dimensional stereotactic surface projection and easy z-score imaging system (eZIS), had been developed.^[5–7]^ These software analyzed the patient's images by voxel-to-voxel comparisons with those from age-matched normal database, and then generated z-score images as a more objective and reliable assessments of functional abnormalities.

In this report, we presented a rare case of superficial siderosis (SS) with severe atrophy of anterior cerebellar lobe, which was initially missed by visual interpretation of the brain perfusion SPECT images but was evidently demonstrated on the result of eZIS analysis.

## 2 Case report

**Figure 1:**
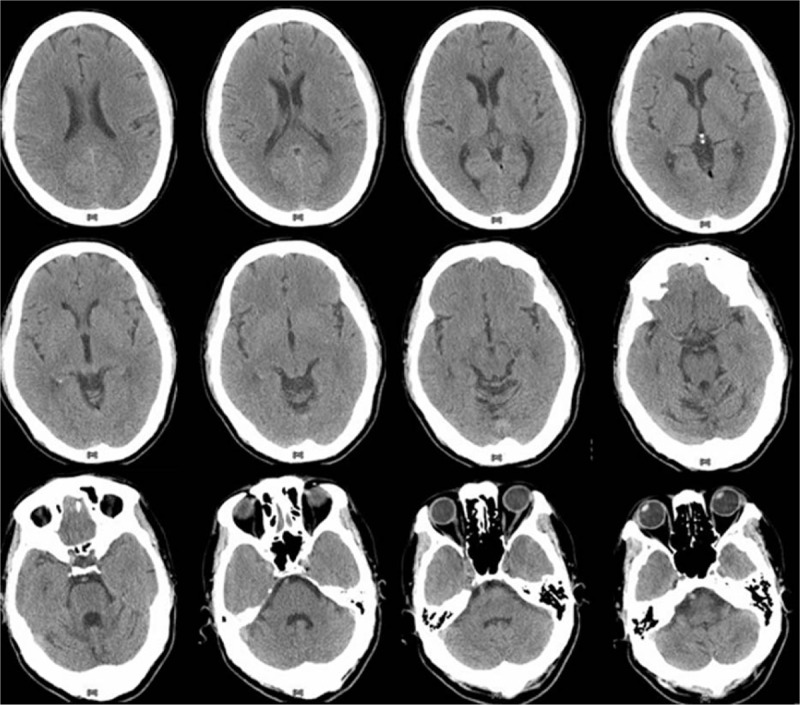
Brain computed tomography scan showed only some low attenuations in right cerebellum, favoring prior sequela lesion.

A 64-year-old male was initially presented with dizziness with spin sensation on and off for 2 days, and then he was sent to emergency room due to further development of nausea and vomiting. Brain CT scan was ordered for assessing possible cerebral vascular accident; however, only some low-attenuation areas were noted in right cerebellum, favoring prior sequela lesion (Fig. 1).

**Figure 2:**
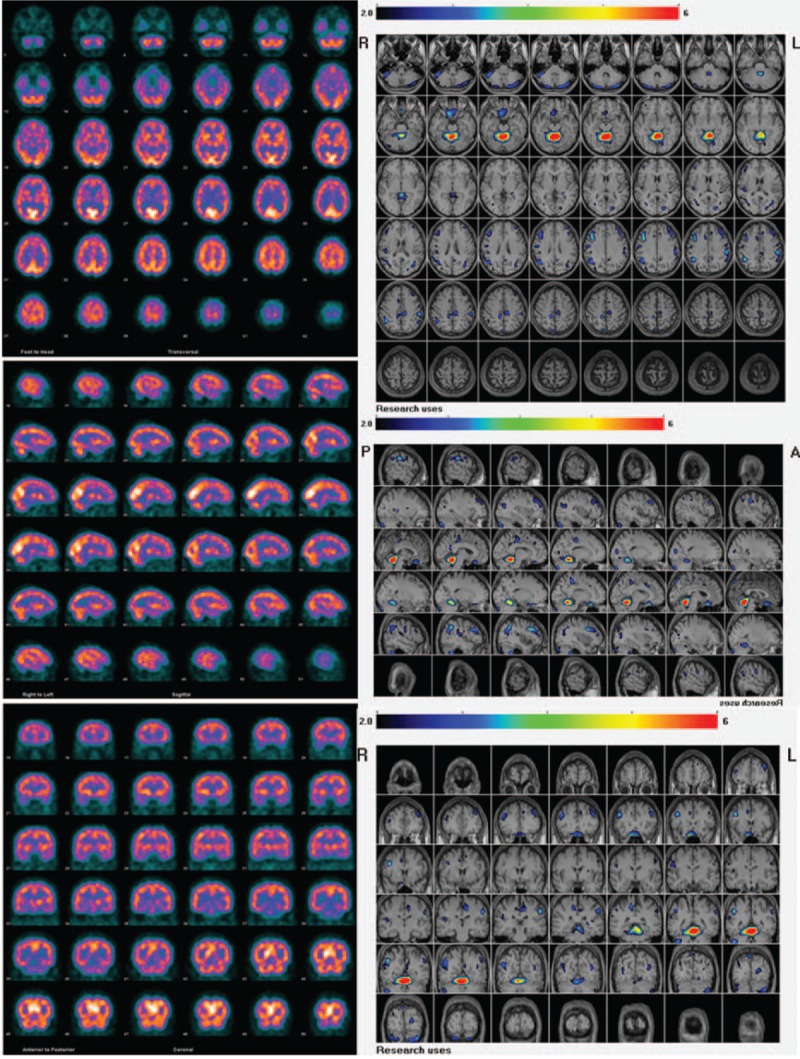
On conventional display of Tc-99m ECD brain perfusion single photon computed tomography (SPECT) (left column), no remarkable abnormality could be identified on SPECT images by readers of more than 5 experienced nuclear medicine physicians. The results of easy z-score imaging system analysis showed remarkable hypoperfusion at anterior lobe of cerebellum, in which red color on the fused magnetic resonance imaging template means Z-score higher than 6 (right column). In addition, mild grade of hypoperfusion in superficial areas of bilateral cerebellum, bilateral frontal, and parietal lobes (blue, green, and yellow colors means Z-score higher than 2 but less than 6).

Reviewing the patient's past medical history, there were only essential hypertension and type-2 diabetes under regular medical control. Lab tests were all normal except for the only abnormality of hyperlipidemia. Neurological examinations revealed multidirection nystagmus, bilateral hearing impairment, wide-based gait, and dysarthria. Tc-99m ECD brain perfusion SPECT was performed for evaluating occult cerebral ischemia, infarction, and/or degeneration. However, no remarkable abnormality could be identified on conventional display of SPECT images. Surprisingly, further software analysis by “eZIS” showed some remarkable hypoperfusion in the anterior lobe of cerebellum (Fig. 2). In addition, mild grade of hypoperfusion was noted in superficial areas of bilateral cerebellum, bilateral frontal, and parietal lobes (blue, green, and yellow colors mean z-score higher than 2 but less than 6), along with the spaces of cerebrospinal fluid (CSF).

**Figure 3:**
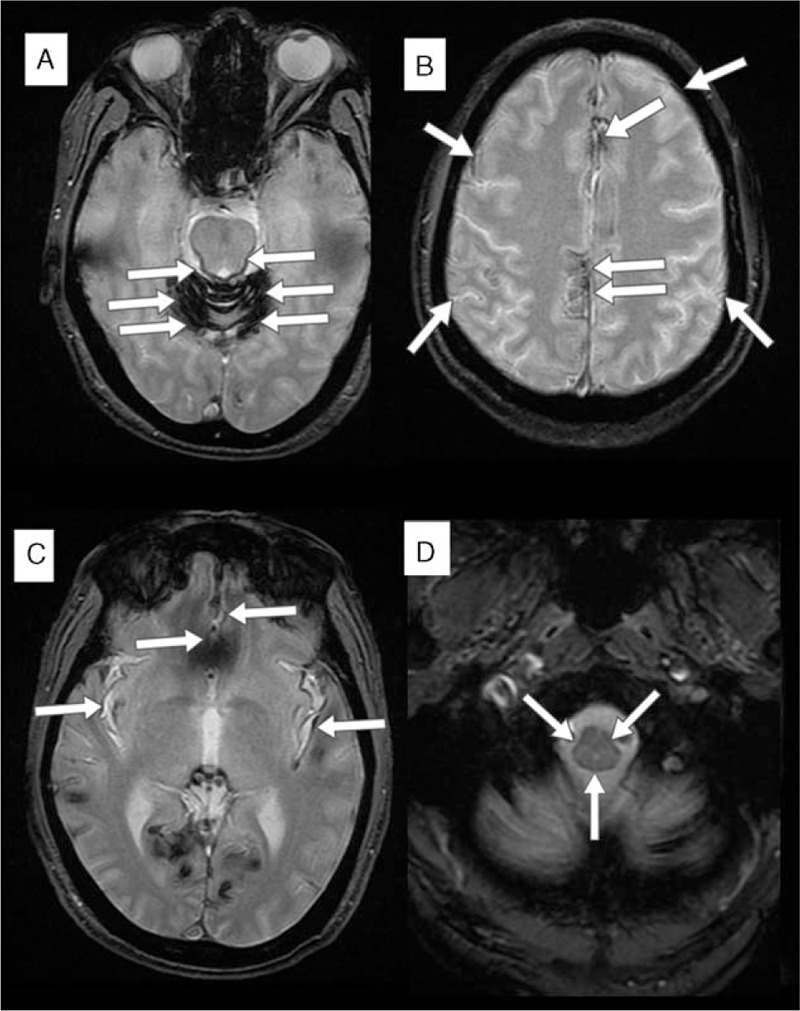
Axial T2-weighted brain magnetic resonance imaging showed diffuse hypointensity signals (white arrows) along with the cerebrospinal fluid spaces, most severe in the cerebellar folia (A), and also around pons (A), higher interhemispheric fissure (B), lower interhemispheric fissure and Sylvian fissure (C), and spinal cord (D).

In order to elucidate the nature of hypoperfusion abnormalities shown on SPECT, brain MRI was performed for further correlation. The T2-weighted images showed diffuse hypointensity signals along with the CSF spaces, most severe in the cerebellar folia (Fig. 3A), and also around pons (Fig. 3A), interhemispheric fissure (Fig. 3B), lower interhemispheric fissure, Sylvian fissure (Fig. 3C), and spinal cord (Fig. 3D), consistent with typical appearances of hemosiderin deposition. In addition, remarkable atrophy of anterior cerebellar lobe was noted, explaining the reason of severe hypoperfusion shown on brain SPECT. In combinations of all the clinical and imaging findings, a final diagnosis of SS was made. Because this report just reviewed previous data and did not involve any human trials, there is no need to conduct special ethic review, and the ethical approval is not necessary. Informed consent was signed by the patient for the publication of this report and its related images.

## 3 Discussion

The major finding of this report is that the automated software of eZIS for analyzing brain SPECT is very useful for enhancing the diagnosis of hypoperfusion abnormalities located at an uncommon region due to a rare disease. Unawareness of the findings of MRI, we ever tested more than 5 experienced nuclear medicine physicians with the conventional displays of SPECT images, including transaxial, sagittal, and coronal views. Unfortunately, no one could correctly identify the severe hypoperfusion abnormality at anterior cerebellar lobe.

As a statistical parametric analysis program, eZIS was developed by Matsuda et al in the National Center for Neurology and Psychiatry (NCNP) of Japan.^[6]^ Using the age-classified normal ECD database as the reference, eZIS has become the most widely used software for analysis of Tc-99m ECD brain SPECT in Japan.^[8,9]^ After normalization to the normal brain ECD template and global or cerebellar mean values, this program performs voxel-by-voxel z-score analysis (z-score = [control mean – individual value]/control standard deviation), and then converted the z-score values into images overlaid on MRI template. Although eZIS provided a reference normal database of ECD SPECT images with high quality and large numbers of healthy volunteers, other institutes may be able to use this normal limit properly because of the variations in image characteristics secondary to different equipment and physical correction algorithms. For correcting the image differences between centers, eZIS allowed the users to convert the images according the 3-dimensional conversion map by dividing the brain phantom images acquired on users’ scanner and NCNP.^[10]^

SS is a rare disease resulting from hemosiderin depositions along the leptomeninges of brain and spinal cord but no history of symptomatic subarachnoid hemorrhage.^[11]^ The major clinical presentations of SS are progressive cerebellar dysfunction (gait ataxia) and bilateral sensorineural hearing loss. The pathological process is believed due to chronic occult subarachnoid bleeding; however, the source of bleeding usually cannot be identified. The assessment and diagnosis of SS could be made by MRI, characterized by the findings of hypointensity signals due to hemosiderin depositions on the pial and epdendymal surfaces, especially the locations of brain stem and cerebellum.

In addition to the anatomy-oriented imaging modality of MRI, the findings of SS on functional imaging, such as positron emission tomography (PET) and SPECT, had ever been rarely reported. In 1994, the investigators in Japan used I-123 IMP (Nihon Medi-Physics, Tokyo, Japan) brain SPECT to evaluate a case of idiopathic SS and found hypoperfusion in the cerebellum and frontal lobe where hemosiderin was heavily deposited.^[12]^ This was so far the only case that the findings of SS on brain perfusion SPECT were reported in the medical literatures. Regarding the findings of SS on PET, Yamazaki et al first reported 2 cases of SS in 1995 and found both cases had reduced cerebral blood flow and cerebral oxygen metabolism in the basal temporal lobes.^[13]^ Also in Japan, the investigators reported a case of SS about the results of long-term imaging follow-up with CT, MRI, and PET.^[14]^ They found that the progressive reduction of cerebral blood flow and oxygen metabolism in the brain stem, cerebellar hemispheres, and temporal lobes were noted in the regions with marked depositions of hemosiderin on MRI.

Using eZIS for analyzing brain perfusion SPECT, the observed cerebellar hypoperfusion had also been found helpful for enhancing the diagnosis of other conditions in addition to SS. Waragai et al^[15]^ found a specific hypoperfusion pattern on eZIS analysis of SPECT in the cerebellum and pons in patients with multiple system atrophy and in the cerebellum in cortical cerebellar atrophy (CCA). The study of Watanabe et al^[16]^ showed that cerebellar perfusion was decreased in patients with CCA and olivopontocerebellar atrophy (OPCA), but eZIS analysis further demonstrated that hypoperfusion at the brain stem and cerebellar nucleus in OPCA group was more significant than in CCA group. For studying the correlation of neuropsychology test performance and SPECT perfusion, Baker et al^[17]^ found that cerebral small vessel disease also might be able to cause hypoperfusion in cerebellar region shown on eZIS analysis. In the case report of Nanri et al, SPECT-eZIS was found useful for detecting cerebellar hypoperfusion in a patient with antigliadin-antibody-positive cerebellar ataxia.^[18]^

In conclusion, the presented case demonstrates the unique value of eZIS analysis of Tc-99m ECD brain SPECT on identifying the severe hypoperfusion in anterior cerebellar lobe and the trivial hypoperfusion in superficial regions of cerebral cortices in a rare case of SS. As an objectively software-assisted tool for ECD images, more clinical experiences are still needed to confirm the incremental value of eZIS in enhancing the diagnosis of neurological diseases.
